# Feasibility and Perception of Using Text Messages as an Adjunct Therapy for Low-Income, Minority Mothers With Postpartum Depression

**DOI:** 10.2196/mental.4074

**Published:** 2015-03-16

**Authors:** Matthew A Broom, Amy S Ladley, Elizabeth A Rhyne, Donna R Halloran

**Affiliations:** ^1^ SSM Cardinal Glennon Children's Medical Center Department of Pediatrics Saint Louis University School of Medicine St. Louis, MO United States; ^2^ Albert Gnaegi Center for Health Care Ethics Saint Louis University St. Louis, MO United States; ^3^ Center for Outcomes Research Saint Louis University St. Louis, MO United States

**Keywords:** short message service, health care disparities, mobile health, mental illness

## Abstract

**Background:**

Postpartum depression (PPD) is the most common medical problem among new mothers that can have a negative impact on infant health. Traditional treatments are often difficult for low-income mothers to complete, particularly given the numerous barriers families face.

**Objective:**

Among low-income, primarily racial, and ethnic minority mothers with postpartum depression, our aim was to evaluate (1) the feasibility of sending supportive text messages, and (2) the perception of receiving private, supportive text messages for postpartum depression.

**Methods:**

Mothers found to be at risk for postpartum depression received supportive text messages four times weekly for 6 months in addition to receiving access to traditional counseling services based within an academic pediatric office. Feasibility was evaluated along with cellular and text messaging use, access, and perception of the message protocol. Perception of the message protocol was evaluated at study completion via a Likert scale questionnaire and open-ended qualitative survey.

**Results:**

In total, 4158/4790 (86.81%) text messages were successfully delivered to 54 mothers over a 6-month period at a low cost (US $777.60). Among the 96 scripted messages, 37 unique messages (38.54%) allowed for a response. Of all sent messages that allowed for responses, 7.30% (118/1616) were responded to, and 66.1% of those responses requested a call back; 46% (25/54) of mothers responded at least once to a text message. Mothers felt that messages were easily received and read (25/28, 89%) and relevant to them personally (23/28, 82%). Most shared texts with others (21/28, 75%).

**Conclusions:**

Text messaging is feasible, well-accepted, and may serve as a simple, inexpensive adjunct therapy well-suited to cross socioeconomic boundaries and provide private support for at-risk mothers suffering from postpartum depression.

## Introduction

Postpartum depression (PPD) is the most common medical problem new mothers face, occurring in 13%-20% of women at some point after birth [1,2]. It has significant effects on children’s behavior: higher rates of infant behavioral problems, impaired social development, delayed cognitive development, and insecure attachment patterns [3].

For low-income and racial and ethnic minority women, the rates of PPD may even be higher [4]. They are also less likely to be diagnosed with, seek out, and/or receive assistance for their symptoms [4]. Traditional barriers to care include a lack of access to medical or counseling services, transportation issues, and a strong stigma against the receipt of mental health therapy [5,6]. For providers, it can be extremely difficult to transcend these barriers and effectively connect with patients.

Short message service (SMS) or text messaging is now a ubiquitous form of communication throughout the world [7]. Texting is inexpensive, and most patients living in poverty have cell phones with texting capability, making it very appealing to underserved minorities [7-9]. Texting requires no transportation and allows patients to receive private, specific messages, which may help reduce the stigma surrounding the receipt of mental health treatment among urban minorities. Daily communication via SMS has the potential to reinforce the therapeutic bond between counseling sessions [10]. Few studies have evaluated using texting as an adjunct therapy for depression. Recently two groups have evaluated the feasibility, patient acceptability, and cost-effectiveness of texting depressed patients, both highlighting the ability of texting to transcend all socioeconomic barriers to care [10,11]. We sought to evaluate the feasibility and patient perception of sending text messages to low-income, primarily African-American mothers with PPD, a previously unstudied patient demographic.

## Methods

### Participants and Setting

This was a prospective pilot study conducted in a single urban, academic pediatric clinic. Between December 2012 and June 2014, screening for PPD occurred as a standard assessment for all mothers presenting at well-child visits between 7 days and 6 months postpartum. English-speaking mothers (N=143) who lived in the city of St. Louis and scored ≥10 (at risk for PPD) on the Edinburgh Postnatal Depression Scale (EPDS) [12] were approached for enrollment by members of the research team. The research team framed participation as an offer of support for PPD. All subjects received similar treatment (cognitive behavioral therapy [CBT]) and were offered participation in text messaging as an adjunct. The study did not have Institutional Review Board approval to collect data on those who chose not to participate, so specific reasons for non-participation are not available. Severity of depression was evaluated with the Beck Depression Inventory-II (BDI-II) [13].

### Study Procedures

Subjects received 4 text messages per week for 6 months, with each subject receiving the same, non-randomized, message script without any repeats. Message scripting existed in isolation from other support services provided by the research team. That is, while messages were developed with CBT themes in mind, they did not specifically coincide with CBT work in therapeutic sessions. Text messages were developed to be supportive in nature, using principles of postnatal education, motivational interviewing, and CBT (Table 1). Messages were developed by the authors (MB, AL, ER) with input from therapeutic staff. Text messages were automated, sent from a bank of 96 messages, and delivered via the Televox Housecall messaging program. Subjects had the opportunity to opt out of text messaging at any time. Some messages allowed for a response in regards to whether or not a subject would like a follow-up phone call; these responses were limited to either a YES or NO. Prior to receiving text messages, all subjects were provided clear guidance that a team member would return the requested call within 24 hours, but that text responses were not to be used for emergent communications. As text responses were limited in scope (YES or NO), subjects were unable to ask any questions or describe any suicidal content to the project team via a text. To further ensure the safety of subjects, a suicide protocol was developed for the practice to be used in the clinic. The protocol was implemented twice over the course of the project.

CBT, an accepted standard of care for PPD, was the primary form of counseling used during the study, and enrolled subjects had unlimited access to therapeutic services [4,14]. In addition, they had access to social work assistance and telephone support services from clinic staff as part of a comprehensive program entitled Happy Mothers, Healthy Families (HMHF).

**Table 1:** Example text messages by category.

Category	Example
Informational	A routine is comforting for babies. It helps them know when things, like eating and napping, will happen.
	Children should see the doctor at 2 weeks, 1 month, 2, 4, 6, 9, and 12 months old. Call XXX-XXXX to make an appointment.
	The best seat for your baby is in a car safety seat for every trip in a car, truck, or van. Do u want HMHF to call? YES/NO
	Some symptoms of depression: crying, anger, anxiety, sleep/appetite changes, guilt, helplessness. Want us to call? YES/NO
	Smoking is unhealthy for you, your baby, and other family members. Call for help, call QUIT NOW at 800-784-8669
Motivational / General support	Recognition for what we do for others may be desirable, but it’s never guaranteed. Celebrate your own accomplishments, big and small.
	Your happiness depends on what you do; what small thing will you do today to create happiness for yourself?
	Bounce off a loss and onto the next win. Win or lose, HMHF is here, do you want us to call you? YES/NO
	Making a plan and moving forward can be scary; the HMHF team is here to support you. Call XXX-XXXX to schedule an appointment.
	Take the first step, no more and no less, and the next will be revealed (Roberts). We are here to help you take that step. Call XXX-XXXX.
Cognitive behavioral therapy (CBT) / Reflection	Today let’s focus on making decisions from the facts not our feelings. What decisions do you have to make?
	We all own responsibility for our feelings; how will you take care of your feelings today? For help making a plan, call XXX-XXXX.
	Focusing on what should be or could be drains energy; focus on what you can achieve. What do you want to achieve? What’s the first step?
	You don’t have choice in what others say or do; You do have choice in how you respond. Call HMHF to talk to the team.
	No one can make you feel inferior without your consent. (E. Roosevelt) What can you do today to make yourself feel empowered?

### Statistical Analysis and Evaluation

In order to define the baseline degree of mobile phone utilization among the study group, cellular and text-messaging use and access were surveyed prior to subjects receiving text messages. Feasibility was defined by the percentage of sent messages successfully received. The perception of the message protocol was evaluated at completion via a Likert scale questionnaire and open-ended qualitative survey. Qualitative comments were pooled. The study was approved by the Saint Louis University Institutional Review Board. Descriptive statistics were used for the evaluation.

## Results

### Demographic and Cellular Use Characteristics

Among the 58 mothers who enrolled (Table 2), 69% (40/58) were between the ages of 20-29 and 83% (48/58) were of non-Hispanic African-American race. The majority (38/47, 81%) had an annual income of <US $25,000 per year. The mean EPDS was 13.57 +/- 3.14 (scores ≥10 indicative of risk for PPD) and the BDI-II was 24.24 +/- 9.73, with scores in the range of 16-25 indicative of moderate depression.

Fifty-four (93%) subjects received text messages. The majority of mothers (98%, 54/55) with cellular phone and texting capability elected to receive text messages while 3 subjects did not have text messaging capability, though each commented that they would have liked to receive messages. Thirty-four (63%) subjects had a smartphone.

**Table 2:** Demographic characteristics of HMHF subjects.

Characteristic		HMHF Moms, n (%)
**Age, years**		
	15-19	5 (8.6)
	20-29	40 (69.0)
	30-39	13 (22.4)
**Race/Ethnicity**		
	Non-Hispanic white	8 (13.8)
	African American	48 (82.8)
	Mixed-Race	2 (3.4)
**Education level (n=48)**		
	Less than high school	12 (25.0)
	High school graduate	10 (20.8)
	Some college	19 (39.6)
	Associate’s	4 (8.3)
	Bachelor’s and above	3 (6.3)
**Income, USD (n=47)**		
	Less than $10,000	25 (53.2)
	$10,000-$14,999	5 (10.6)
	$15,000-$24,999	8 (17.0)
	$25,000-$34,999	3 (6.4)
	Greater than $35,000	6 (12.8)

### Feasibility

**Figure 1:**
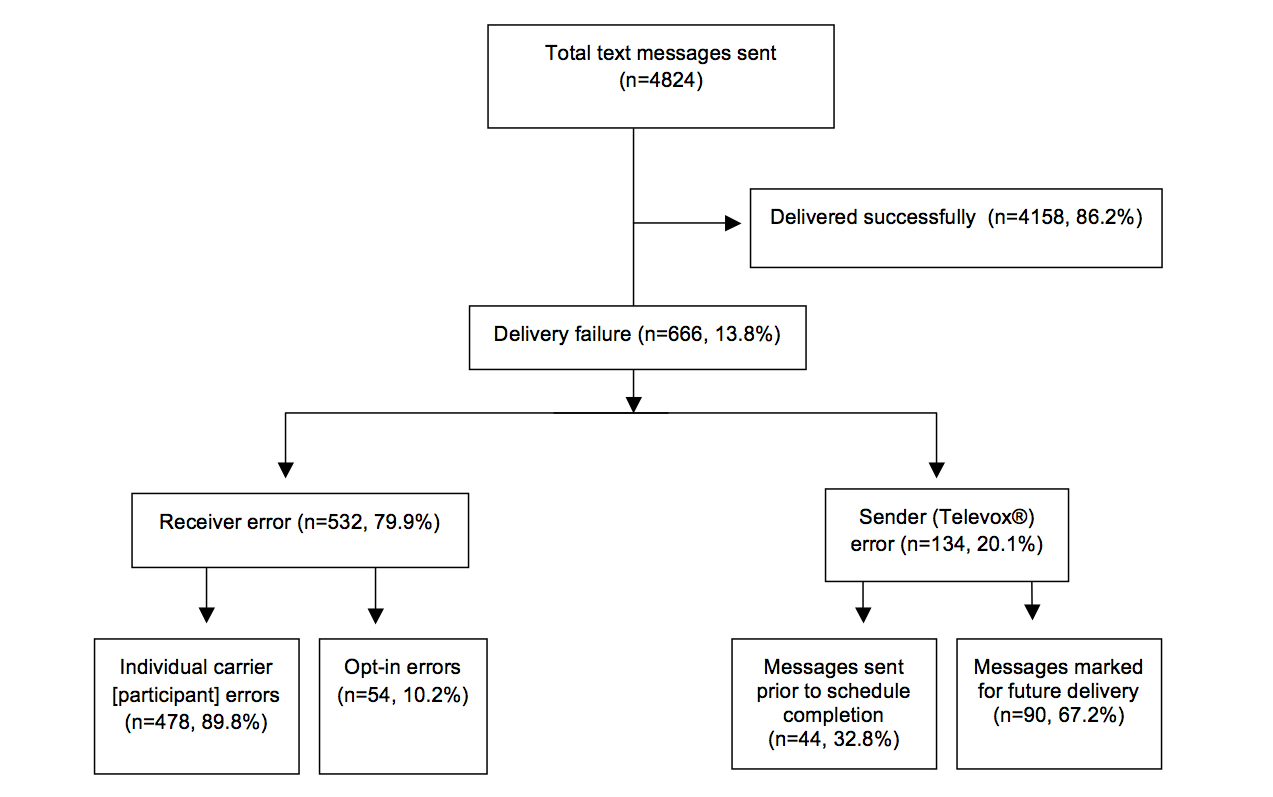
Text messaging feasibility.

A total of 4158 texts were sent successfully with 13.81% (666/4824) experiencing delivery failure. Of those messages not received, 532/666 (79.9%) had a receiver error and 134/666 (20.1%) had a sender error. Among receiver errors, 478/532 (89.8%) were individual carrier (participant) errors (eg, lapse in service, changed number, texting plan did not support short code messages), and 54/532 (10.2%) were opt-in errors, which resulted when a participant accidentally opted out and did not recognize it. Among the sender errors, 44/134 (32.8%) messages were not sent due to Televox stopping those messages prior to completion of the message schedule, and 90/134 (67.2%) messages were marked for future delivery by Televox, denoting that the message may or may not have been sent after the scheduled time (Figure 1).

Among the 96 scripted messages, 26% (25/96) included a call-back number, and 39% (37/96) included a YES/NO prompt to text whether the recipient would like a follow-up call. Of the messages that allowed for responses, 7.30% (118/1616) were responded to, and 66.1% of those responses requested a call back; 46.3% (25/54) of mothers responded at least once to a text message. Total cost for sending all messages during the project was US $777.60 ($14.40/subject; 54 subjects). This cost was based on the price per message sent ($0.15/message; 96 messages).

### Survey Data

Through June 2014, 28 subjects had completed both 6 months of enrollment in addition to an exit survey. Two subjects opted out of text messaging during the 6-month message cycle while the remaining 28 either elected not to fill out an exit survey (n=12), had not yet completed 6 months of study participation (n=8), or withdrew (project team unable to contact after completing message protocol) from the study (n=8). Of those who completed an exit survey (Table 3), the majority agreed (25/28, 89%) that texts were a positive influence on their motivation to change and on their symptoms of depression. Patients felt that messages were easily received and read (25/28, 89%) and relevant to them personally (23/28, 82%). Most mothers shared the texts with others (21/28, 75%).

Regarding qualitative responses, Table 4 details all individual responses for each open-ended question from the exit survey. When asked the best thing about the text messages, the majority of respondents indicated it was their ability to motivate and recalibrate. Subjects appreciated that the messages were positive and encouraging, serving as a call to action, or a reminder of the capability to handle their PPD. By extension, subjects used the messages as a boost in challenging themselves to deal with their depression. The majority of subjects indicated that they would not change anything about the messages, but some subjects wanted the messages to be more customizable both in content and timing.

**Table 3:** Patient perception of messages based on exit survey.

Quantitative survey responses^a^	Average; % agree (n)
Overall, I enjoyed receiving text messages from the Project Team.	4.32; 79% (22)
It was easy to receive and read the text messages from the Project Team.	4.50; 89% (25)
I feel that the text messages made a positive change in my symptoms of depression.	4.32; 82% (23)
I thought the messages were very relevant to me personally.	4.29; 82% (23)
I feel that the text messages were a positive influence on my motivation to change.	4.50; 89% (25)
I liked having the option for an HMHF Project Team member to call me back.	4.52; 89% (25)
I shared the text messages with others.	4.19; 75% (21)

^a^All measured on a 1-5 Likert scale where 1=strongly disagree and 5=strongly agree.

**Table 4:** Qualitative responses from exit survey.

Survey question	Responses
What was the best thing about receiving text messages from the HMHF Project Team? (n=27)	The encouragement (4)
	The most fantastic part about receiving the text messages was that it gave me something to dwell on after a bad situation; it made me think positive (3)
	Knowing that someone was there for me and that [the team] cared (3)
	If I was down, it helped me through the day; they always cheer me up (3)
	Positive messages (3)
	They always seemed to come when I needed them most (2)
	They help (2)
	I liked that they came in the morning to help set the “tone” for the day (2
	The reminded me every day, even just by their presence, that I had tools, resources, and thoughts that improve my depression
	I didn’t like them; they need to be more positive
	You can reply [to the messages] for help
	Receiving the calls
	Too few [messages]
How did you use the messages sent by the HMHF Project Team? (n=26)	Encouraging myself to do better for my child and I (5)
	I would try to make sure I incorporated it into my day (5)
	As a confidence booster and to cheer up. I also shared them with friends and family members (3)
	I would use the message to motivate me (3)
	To gain hope and strength (2)
	I just read them (2)
	I used the messages as an inspiration (2)
	I will reread over it again sometimes
	I started to see it like my daily horoscope; not all days was it completely relevant, but the days it was spot on were awesome
	It [kept] me knowing people care
	I used them as little prayers for me something to look up to
What would you change; what would have been more helpful? (n=26/16)	Nothing (23)
	More inspirational quotes (5)
	The messages that have been helpful are those that apply to “moving forward” in life, paying attention to yourself, having focus (4)
	Not so many (2)
	Anything positive (2)
	It would have been more helpful to choose a topic for the week and relate it to my situation
	I particularly liked the quotes. Perhaps more fact-based messages would have been helpful, only because I tend to respond to statistics and research
	I would send more
	I wish [the messages] came at a specific time of day that I selected
	I wish I [could] still receive them
	I don’t know

## Discussion

### Principal Results

#### Feasibility

Messages were received with a high level of success (86.8% of those sent), and program cost was considerably less than anticipated indicating feasibility. Among low-income populations, consistent cellular service (eg, changes in phone number, plan) can be a significant barrier. However, given the open communication and consistent access to care offered with this project, reliable contact via SMS was not a major issue. Some subjects changed phone numbers during the program; however, based on the frequent clinical interactions at maternal counseling and infant well-child visits, the project team was able to quickly update contact information for the texting program as a team member checked in with subjects at all visits to the clinic. Many subjects who changed phone numbers contacted the project team directly to update their new number so that they would continue to receive text messages without any disruption to the texting program. The desire of subjects to quickly alert the project team to changes in their contact information underscores how much they enjoyed the program and the text messages they received.

#### Perception

Mothers clearly felt comfortable receiving the text messages, thought they were informative and appropriate to them as individuals, and were perceived to make a difference in their depressive symptoms. Many mothers noted that they would have liked to receive more individual, patient-specific information via the messages. The possibility for such a program exists; most securely, it would require mothers to own a smartphone with a password-protected messaging app to minimize the possibility of someone recognizing that the phone’s owner was receiving treatment. Alternatively, patients could sign a Health Insurance Portability and Accountability Act (HIPAA) waiver consenting to the receipt of individualized text messages from their medical provider. Given the stigma and sensitivity surrounding treatment for mental illness, programs interested in detailing specific information about depression or anxiety to recipients should strongly consider password protection via an app or utilizing a screen-lock on the phone.

The willingness to share messages about one’s mental health suggests that recipients were engaged in the messaging process, used the messages as a tool to reach out for help, and may be willing to work against the stigma associated with mental health treatment. This is particularly relevant given the noted disparities in mental health care and access among racial and ethnic minority populations, coupled with the strong, often community-specific stigma regarding the receipt of counseling or support services. Based on the qualitative responses in Table 4, this population of African-American women preferred motivational and inspirational messages and noted a preference to receive more individualized messages. Texting may be an avenue to improve engagement with at-risk mothers and families regarding mental health concerns, which traditionally have been taboo.

The mobile, asynchronous nature of texting offers a notable opportunity to bridge traditional barriers to care (poverty, stigma, transportation, and access) [5,15]. The possibility of combining text messaging and traditional CBT as a new treatment model could easily be adopted in primary care, postnatal, and mental health care settings.

### Comparison With Prior Work

These findings correlate with previous studies suggesting that text messaging is a feasible method of communication in health care and an acceptable tool for behavioral change [16-20]. This research is singular given the study population, study duration, and the underlying disease process. Previous authors have described study strength in the investigation of urban, low-income, African-American mothers, noting the high-risk nature and prevalence of PPD within this demographic [21]. Despite this, no studies have evaluated the feasibility or acceptability of text messaging among urban, low-income predominantly African-American mothers with PPD. Although a few studies have evaluated the use of text messaging as an adjunctive tool for depression and mental illness [10,11,22,23], the authors are unaware of any research evaluating text messaging among women with PPD, particularly over a 6-month period. More notably, there is no published data on whether our study population would even use this potential treatment option for PPD. The impact of PPD on a mother spans beyond what accompanies depression alone, as it has a direct impact on infant care, maternal-newborn attachment, and future child development and behavior [24]. Our findings also acknowledge the importance of considering cultural differences when choosing content for a text messaging program [22], something that has not been evaluated in all texting studies [23]. Given the relevance to maternal and child mental health, the evaluation of an underserved population, and the demonstrated acceptance of receiving and sharing messages about a traditionally highly sensitive disease (PPD), many possibilities for further study remain.

### Limitations

The purpose of the evaluation was to assess feasibility and perceptions regarding message content. Because this study did not evaluate text messaging as an intervention for PPD, our findings underscore the need for a larger, controlled study evaluating the clinical efficacy of using text messages as an adjunct therapy for PPD among low-income African-American women.

Not all subjects completed exit surveys, which affected the overall number of responses in the qualitative survey. As a result, a formal qualitative analysis of the comments was not performed given the limited number of responses.

### Conclusions

Increasing access to mental health services via mobile technology has the opportunity to positively impact infant developmental, behavioral, and attachment outcomes that are linked to PPD. The minimal cost of executing a text messaging protocol creates a viable, scalable, and fiscally responsible option for providing adjunctive support towards the treatment of PPD. As traditional treatments are often unavailable or difficult for low-income mothers to complete, text messaging may serve as a simple, inexpensive adjunct therapy well-suited to cross socioeconomic boundaries and provide private support for at-risk mothers suffering from postpartum depression.

## Abbreviations

CBT: cognitive behavioral therapy
EPDS: Edinburgh Postnatal Depression Scale
HMHF: Happy Mothers, Healthy Families
PPD: postpartum depression
